# Development of an EEG Headband for Stress Measurement on Driving Simulators †

**DOI:** 10.3390/s22051785

**Published:** 2022-02-24

**Authors:** Antonio Affanni, Taraneh Aminosharieh Najafi, Sonia Guerci

**Affiliations:** 1 Polytechnic Department of Engineering and Architecture, University of Udine, 33100 Udine, UD, Italy; aminoshariehnajafi.taraneh@spes.uniud.it; 2 Eurisoft S. P., 33010 Tavagnacco, UD, Italy; info@eurisoft.it

**Keywords:** brain activity, stress measurement, EEG sensor, driving simulators

## Abstract

In this paper, we designed from scratch, realized, and characterized a six-channel EEG wearable headband for the measurement of stress-related brain activity during driving. The headband transmits data over WiFi to a laptop, and the rechargeable battery life is 10 h of continuous transmission. The characterization manifested a measurement error of 6 μV in reading EEG channels, and the bandwidth was in the range [0.8, 44] Hz, while the resolution was 50 nV exploiting the oversampling technique. Thanks to the full metrological characterization presented in this paper, we provide important information regarding the accuracy of the sensor because, in the literature, commercial EEG sensors are used even if their accuracy is not provided in the manuals. We set up an experiment using the driving simulator available in our laboratory at the University of Udine; the experiment involved ten volunteers who had to drive in three scenarios: manual, autonomous vehicle with a “gentle” approach, and autonomous vehicle with an “aggressive” approach. The aim of the experiment was to assess how autonomous driving algorithms impact EEG brain activity. To our knowledge, this is the first study to compare different autonomous driving algorithms in terms of drivers’ acceptability by means of EEG signals. The obtained results demonstrated that the estimated power of beta waves (related to stress) is higher in the manual with respect to autonomous driving algorithms, either “gentle” or “aggressive”.

## 1. Introduction

Over the last few decades, with the rapid growth in technologies and the evolution of Artificial Intelligence (AI), computers have become more pervasive, not only in industries, but also in offices and homes. The words “smart” and “intelligent” are commonly used today to label digital devices. Smartphones, once considered luxury items, have become a necessity for many. Similarly, smart and autonomous cars will lead the automobile markets in the near future. They can have different levels of Advanced Driver Assistance Systems (ADASs), from no control (Level 0) to fully autonomous vehicles (Level 5) [1]. They are expected to be safer and to reduce the traffic on the roads thanks to Vehicle-to-Vehicle (V2V) and Vehicle-to-Infrastructure (V2I) communications [2]. In order to have a smooth transition toward autonomous vehicles and, in general, to all AI devices and services, the human–machine communication should be studied and developed as well. Recognizing human emotions, their experience, and their levels of comfort and stress while using AI devices or services can help to calibrate the functions to be performed and the decisions to be made by AI accordingly. For example if autonomous vehicles consistently make automated choices that follow passengers’ expectations, they could create a higher level of trust, which is fundamental to promoting their acceptance [3], while failing to do so would instead lead to mistrust and stress.

Stress is a physiological and psychological reaction to frightening situations. Normally, it occurs when the perceived events are different from the expected ones [4]. Brain neurons release many chemical messengers called neuropeptides in response to stress [5]. As a result of this action, the secretion of a series of hormones including adrenaline and cortisol will be enhanced to prepare the individuals for their fight or flight response by giving them extra strength and speed to either fight or run away from danger. When the fight or flight response begins, the Autonomic Nervous System (ANS) will be activated, causing instantaneous and strong changes in the body. The ANS controls various automatic routine activities of the body including digestion, heart beat, blood pressure, and body temperature. The ANS manages the fight or flight response of the body through two branches: the sympathetic and parasympathetic nervous systems. The sympathetic nervous system initiates the fight or flight response as an automatic reaction to prepare the body to face the possible threat or to support eventual pain. The parasympathetic nervous system is responsible for moving the body back to its balance state after the termination of stressful situations. During the fight or flight response, the sympathetic nervous system increases several blood factors such as flow, pressure, sugar, and fats; moreover, it tenses muscles, dilates pupils, and increases the breathing rate, perspiration, and brain neural activities [6].

In order to measure stress, the mentioned physiological changes can be evaluated through wearable biosensors. Various methods and biological sensors have been proposed and presented in the literature. In a previous study [7], we presented our designed Skin Potential Response (SPR) sensor, which is able to measure the electrical activity of the sympathetic nervous system on the palms, where sweat glands are situated, based on the Electro-Dermal Activity (EDA) principle. In another study [8], we presented an adaptive filters algorithm to remove hand movement artifacts from SPR signals. In [9], we monitored the stress level of drivers during manual and autonomous driving scenarios on a professional simulator using our designed SPR sensor. In [10], the authors measured EDA signals to identify stress and anger in 20 subjects while driving on a simulator. In [11], pupil diameter signals were employed for stress measurement of drivers during a simulated driving experiment. In [12], several bio-signals such as Electrocardiogram (ECG), Electromyogram (EMG), Galvanic Skin Response (GSR), Heart Rate (HR), and Respiration Rate (RESP) were evaluated with Machine Learning (ML) algorithms to classify stress in drivers. The study concluded that the ECG achieved the highest stress classification accuracy.

The brain is the core of the nervous system; therefore, complex patterns of neural activities packed with valuable physiological information can be recorded from the surface of the head by Electroencephalogram (EEG) sensors. EEG is able to measure the currents that flow within brain neurons on the scalp. Neural oscillations or brain waves are the result of synchronized electrical activities of large groups of neurons and are generally categorized by their frequency, as shown in Table 1 [13,14,15].

**Figure 1:**
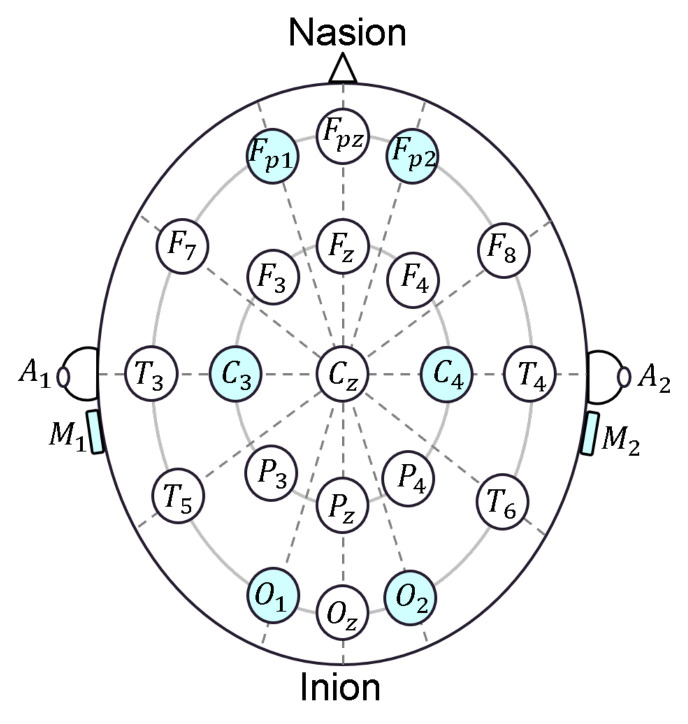
Electrode placement according to the 10/20 system. The electrodes used in the developed headband are highlighted in blue.

The electrical signals detected by the scalp electrodes are very weak, usually from 10 μV to 100 μV, because between the electrode and neuronal layers, there are the skin, skull, and several other layers that significantly attenuate the signals. Electrode placement on the scalp is defined by the International 10/20 Standard [16]. Electrodes are labeled according to the different brain areas: Frontal (F), Central (C), Temporal (T), Posterior (P), and Occipital (O); A and M are the ear and Mastoid electrodes, respectively; they are usually used as references. The left side of the head is indicated by odd numbers and the right side by even numbers [13]. Electrodes and their placement are shown in Figure 1.

The EEG signal can be distorted from the original one to a higher amplitude and different shapes by unwanted physiological signals such as cardiac and muscle activities, eye movements and blinks, or by external sources such as AC power line noise (50/60 Hz), impedance fluctuation, cable movements, and broken wire. These distortions are called artifacts [13]. Artifacts from external sources can be minimized with more precise experimental setups and strict recording procedures, whereas physiological artifacts are usually removed or corrected from the acquired signal during the pre-processing steps. The motion artifact that arises on low-level bio-signals is a well-known problem [17,18,19] when the subject is performing physical activity. Various artifact-removal techniques have been presented in the literature; different methods are ideal for different applications, but Independent Component Analysis (ICA)-based algorithms are the most popular because they can deal with almost all types of EEG signal artifacts [20].

EEG sensors have multiple advantages: they are non-invasive, low cost, and easy to set up; they acquire real-time data with high temporal resolution; they are known to be able to detect different brain activities, mental states, and emotions of an individual such as stress [15,21,22,23], attention [24], drowsiness [25,26,27,28], confusion [29], focused, unfocused, and drowsy [30], joy, anger, sadness, and pleasure [31], and happiness, sadness, and relaxed [32]. The use of EEG to analyze drivers’ stress has attracted the attention of researchers in recent years along with the development of autonomous cars. In [33], the study proposed a combined fuzzy and case-based reasoning (Fuzzy-CBR) classification approach to identify the stress or relaxed states of drivers using EEG signals. The proposed method scored a classification accuracy of 79%. In the research undertaken in [34], EEG and ECG signals in addition to electric vehicle data were acquired from 40 drivers during real driving tasks to classify drivers’ stress level. It was shown that the stress level in the drivers was not only affected by the environment conditions such as the road, traffic, and driving duration, but also by individual patterns. In [35], the researchers used three Machine Learning (ML) algorithms: Support Vector Machine (SVM), Neural Network (NN), and Random Forest (RF), to classify EEG signals acquired from 50 subjects while driving in stressful and calm simulated driving setups. An overall accuracy of 97.9% was achieved by a decision fusion method that combined the three models at the decision level. In [36], the authors used the real road driving information of 28 subjects to train an SVM model. The training data were labeled by EEG recordings of the drivers, which were divided into “normal” and “overload” categories. The study achieved a 74.3% classification accuracy by the combination of various driving and vehicle data.

When measuring the stress in individuals, it is of paramount importance to collect data by different methodologies of bio-signal acquisition; in the past, for instance [37], we developed a sensor system that integrated the ECG and EDA signals with a unique timebase thanks to the fact that we designed all the sensors from scratch: in this way, it was possible to have the full control of the raw data, and we could align them with an accuracy of up to 50 ms. Using the same approach, here, we designed from scratch an EEG headband that allows the full control of the raw data and allows the integration of EEG, ECG, and EDA with a unique timebase. Using commercial sensors instead, the users cannot access the raw data, and the time alignment has poor accuracy, since every sensor sends data to its proprietary software, which does not interact with other proprietary control panels; thus, the user must manually insert markers in every graphical user interface, losing repeatability on time alignment.

Beyond the great advantage of full control of the raw data and time alignment, the headband that we present has the advantage of a full characterization, described in Section 3.1; in most commercial EEG sensors, in fact, the specifications do not report the linearity of the sensors; sometimes, the resolution is reported in terms of the number of bits of the Analog to Digital Converter (ADC), but the quantization step size is not provided; thus, the resolution of the voltage readout is not provided; this means that the user is not allowed to know how accurate the readout (and therefore, the signal processing) is. As an example, we report in Table 2 a comparison obtained from commercial devices’ technical specifications and from [38].

Table 2 shows that the presented sensor manifests high performances in terms of battery life, very high resolution, and high linearity. Moreover, the cost of the developed sensor is by far lower than the commercial devices.

Even if linearity and/or measurement error are not provided for commercial devices, most of the scientific literature acquires EEG signals using commercial sensors, and the data are then processed with ML algorithms in order to classify the stress in drivers, especially using driving simulators. Some studies used the help of other bio-sensors to categorize and label the acquired EEG signals [33,34] (again, we point out the importance of a precise time alignment between different sensors); some other studies relied on the drivers’ self-report [35]; other studies classified the mental activity using an arbitrary threshold on the EEG signal level and labeling the categories on the basis of threshold trespassing [36]. In this study, we adopted the analysis of the beta activities to identify the stress in drivers from the acquired EEG signals, and this method is well known in the literature [39,40,41,42]. Few studies have designed and developed EEG sensors with various architectures. For example, in [43], the authors developed an EEG sensor using three prefrontal electrodes, evaluated the signal quality, removed ocular artifacts, and identified the chronic stress using EEG dynamic features; however, no characterization of the sensor’s measurement error, as defined in [44], was provided.

In this study, we designed an EEG sensor from scratch, which provided us more flexibility in the choice of electrodes’ location, montage, and references that normally are not modifiable in commercial sensors. Then, we set up the measurement procedure to experimentally characterize the developed sensor, as described in Section 3.1, in order to quantify the measurement error on the acquired signals, accordingly to [45]; this aspect is very important in order to understand the performances of the sensor, and as previously said, this quantity is often missing in manuals of commercial devices. Finally, we employed our designed sensor to detect the stress in subjects while driving on an advanced simulator setup, which included a movable platform with three degrees of freedom, a steering wheel, and pedals. Furthermore, in order to provide a more realistic simulation, our participants were equipped with a Virtual Reality (VR) headset.

This work is the extended version of the paper presented in [46]; in that paper, we showed a preliminary and partial characterization of the developed sensor, and we provided the results obtained from a single subject under test, in order to verify whether the headband was able to discriminate brain waves’ properties under different driving conditions. In the present paper, we provide the complete metrological characterization of the sensor, and we set up an extended experiment, which involved ten volunteers on a driving simulator. The volunteers had to cope with three different vehicles (one with manual driving, two with different autonomous algorithms) while the headband was acquiring the EEG signals during the tests.

The paper is organized as follows. Section 2 provides the design procedure of the six-channel EEG circuit and the description of the experimental setup to acquire signals from drivers on the simulator. Section 3 shows the experimental results obtained from the characterization and from the post-processed data captured during driving. In Section 4, we discuss the obtained results, and in Section 5, we draw the conclusions.

## 2. Materials and Methods

This section is organized into two parts: in the first part, we describe the design of the developed EEG headband with the considerations of the required technical specifications; in the second part, we describe the experimental procedure followed to acquire the EEG signals of ten volunteers during driving on a simulator.

### 2.1. EEG Headband Design

**Figure 2:**
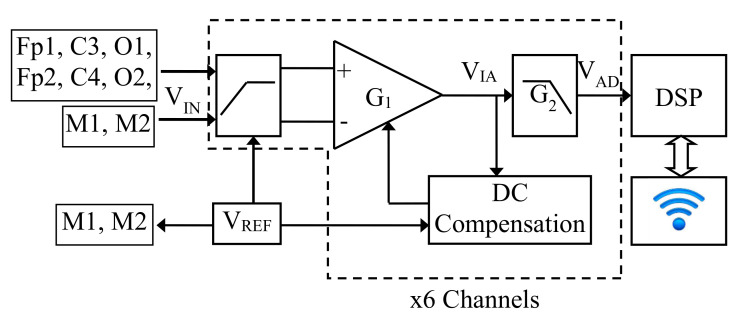
Block diagram of the EEG headband circuit; the dashed box represents the analog front-end of each channel.

The developed headband (whose scheme is shown in Figure 2) has six dry comb electrodes located at Fp1, Fp2, C3, C4, O1, and O2 positions accordingly to the International 10/20 system with common reference montage. The quantity $V_{IN}$ indicated in Figure 2 is the low-level differential voltage between each electrode and the reference electrodes M1 and M2. $V_{IN}$ is properly conditioned by amplifying and filtering accordingly to the specifications described in Section 2.1.1; the high-level signal $V_{AD}$ is acquired by the Analog to Digital Converter (A/D) on board a Digital Signal Processor (DSP) and is sent, via a Universal Asynchronous Receiver Transmitter (UART), to a WiFi module, which transmits the data to a laptop where a custom Graphical User Interface (GUI), described in Section 2.1.3, was developed for data saving and signals’ real-time plotting.

The EEG headband is supplied by a single 850 mAh Lithium Polymer (LiPo) cell battery; since the headband current consumption is 85 mA (the main consumption is due to the WiFi module during data transmission), the chosen LiPo cell allows 10 h of continuous transmission: thus, the battery life is by far longer than typical EEG acquisitions, which are, roughly, 1 h long. A buck DC–DC converter (not shown in Figure 2) provides a +3.3 V supply voltage for the entire circuit; the reference voltage $V_{REF}$ = 1.65 V is generated using a linear voltage reference and is applied to the M1 and M2 reference electrodes.

**Figure 3:**
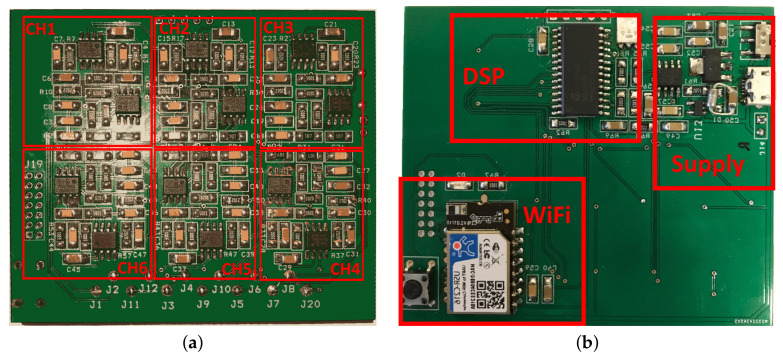
EEG circuit realization on PCB: (**a**) top layer, (**b**) bottom layer. Red rectangles show the blocks of the circuit.

Figure 3 shows the printed circuit board of the sensor; the outer dimensions of the circuit are 75 ${\mathrm{mm}}^{2}$× 65 ${\mathrm{mm}}^{2}$; red rectangles and labels indicate the blocks’ layout in the circuit. In particular, the six-channel analog front-ends are located in the top layer, while the power supply, $V_{REF}$ generation, and WiFi module are in the bottom layer in order to separate the low-level signals from digital signals.

#### 2.1.1. Analog Section

Referring to Figure 2, the analog front-end of each channel measures low-level differential voltages and, through a proper signal conditioning, provides a high-level voltage, which can be acquired by the A/D converter on board the DSP. As a first step, the common mode DC voltage that may be present on the scalp must be removed, so a couple of passive first-order high-pass filters were posed at the circuit input stage. The input impedance of the filters was set to 100 MΩ in the passband; in this way, the load uncertainty results are lower than 1%, considering that the skin impedance is in the order of 1 MΩ. Connected to the filters’ output, there is the instrumentation amplifier (IA, MCP6N11 from Microchip), which was designed with a gain $G_{1}=680$; the IA output is connected to an active second-order low-pass filter (Sallen–Key topology) whose gain is $G_{2}=6.6$ followed by a passive first-order low-pass filter. The overall gain $G=G_{1}\cdot G_{2}=4488$, so the EEG signals, in the range ±350μV, are amplified to 3.3 $V_{PP}$.

The block named “DC Compensation” in Figure 2 compensates the DC non-idealities of the IA (such as the offset current and offset voltage); it performs the integration of the voltage difference $V_{IA}-V_{REF}$; thus, $V_{IA}$ at steady-state follows $V_{REF}$. This block in the feedback loop results in a high-pass filter with a single pole posed at the inverse of the integrator time constant $\tau_{I}$ [37,47]; this quantity was set by design equal to the time constant of passive high-pass filters $\tau_{HP}$. The anti-alias, third-order, low-pass filter was designed to have three coincident poles at time constant $\tau_{LP}$. The transfer function of the analog section for each channel results, in the Laplace domain, as:

(1) $$V_{AD}\left(s\right)=V_{REF}+\frac{G_{1}\cdot G_{2}\tau_{HP}^{2}\cdot {\left(s\right)}^{2}}{{\left(1+\tau_{HP}\cdot s\right)}^{2}{\left(1+\tau_{LP}\cdot s\right)}^{3}}\cdot V_{IN}\left(s\right)$$

Designing $\tau_{HP}\equiv \tau_{I}=0.33$ s and $\tau_{LP}=2.7$ ms, the analog section behaves as a bandpass amplifier having a lower cutoff frequency of 0.8 Hz (lower slope +40 dB/dec), an upper cutoff frequency of 44 Hz (upper slope –60 dB/dec), and a center-band gain of 72 dB.

#### 2.1.2. A/D Conversion, DSP, and Data Transmission

The signals $V_{AD}$ are connected to the analog inputs of the DSP (DSPIC30F3013 from Microchip), which operates at 8 MIPS and has an on-board 12 bit A/D converter. Considering the designed gain discussed in the previous subsection, we would obtain a resolution on $V_{IN}$ of $3.3/\left(G\cdot 2^{12}\right)\approx$ 200 nV, approximately. In order to obtain higher resolution, we oversampled the signal by a factor of 16. The desired sampling rate for transmitting signals was 200 Sa/s; we oversampled at a rate of 3200 Sa/s, and we built the output datum as the sum of 16 samples; in this way, the signal range was increased by a factor of 16, and the noise was increased by a factor of 4; naming *S* the signal and *N* the noise, we have in fact:

(2) $$S=\sum_{i=1}^{16}S_{i}≅16\cdot S_{i}\;,\;N=\sqrt{\sum_{i=1}^{16}N_{i}^{2}}≅4\cdot N_{i}\;\Rightarrow \;\frac{S}{N}=4\cdot \frac{S_{i}}{N_{i}}$$

In this way, we increased the signal-to-noise ratio by a factor of four, corresponding to an increase of the resolution of 2 bit, thus obtaining an effective A/D resolution of 14 bit corresponding to 50 nV, approximately.

The 14 bit data were then sent at 200 Sa/s with a baud rate of 115,200 bps to the low-power WiFi module (USR IOT C216), which sends TCP packets to the laptop.

#### 2.1.3. Software Description

**Figure 4:**
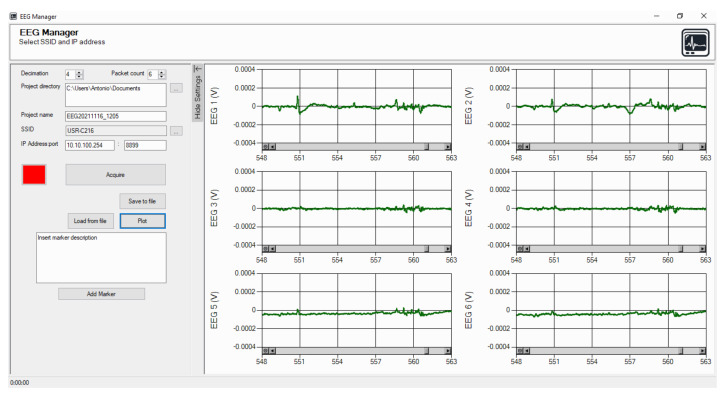
Control panel developed in the .NET environment for data acquisition and real-time plot of the six EEG channels.

We developed, using the .NET framework, a GUI (shown in Figure 4) responsible for acquisition, visualization in real time, and saving of the data transmitted by the EEG headband. The GUI implements also an optional, second-order, IIR notch filter for the rejection of the power line frequency.

The panel was designed in the .NET framework since this tool allows a fast and easy design of Graphical User Interfaces (GUIs). The GUI communicates with the EEG headband via TCP, extracts the data from the received packets, plots the six signals in real time, and allows the insertion of graphical markers with optional comments if the user needs to annotate which kind of stimuli are received by the subject under test. On the left of the GUI, there are several controls where the user sets the SSID of the wireless network and the folder path where data are saved. The button “acquire” starts the connection to the laptop and the plot of the six traces in real time.

### 2.2. Acquisitions of EEG Signals on a Driving Simulator

We tested the developed headband acquiring EEG signals from ten volunteers during driving on the driving simulator available in the BioSensLab at the University of Udine [48].

**Figure 5:**
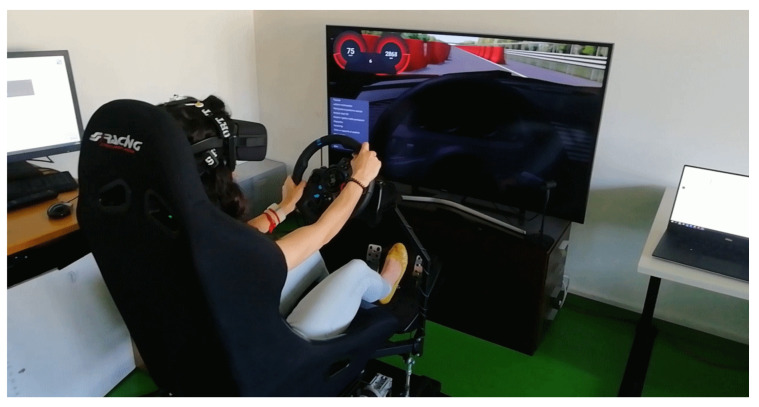
The driving simulator available in our laboratory at the University of Udine.

The driving simulator (Figure 5) is composed of a desktop PC, a moving platform with three degrees of freedom (P3 from DOFReality), a steering wheel with pedals (Logitech G29), and a Virtual Reality (VR) (Oculus Rift) headset [49,50,51]. As the simulation software, we used DriveSim20 by VI-Grade [52]; this software allows the insertion of obstacles on the road, the change of vehicle dynamics, and the replay of saved simulations.

#### 2.2.1. Data Acquisition

Ten volunteers (seven males, three females, aged 29±5) from the University of Udine participated in the experiment; their average driving experience was 11±5 y using cars with a manual transmission. We chose participants among students and employees of our institution that had no previous experience with driving simulators; in this way, we avoided previous VR exposure possibly affecting the response to the virtual driving experience. After the introduction to the EEG headband, VR headset, simulator platform, and the procedure of the experiment, they were invited to sit on the simulator platform wearing the developed EEG headband together with the VR headset and perform the experiment as described below.

**Figure 6:**
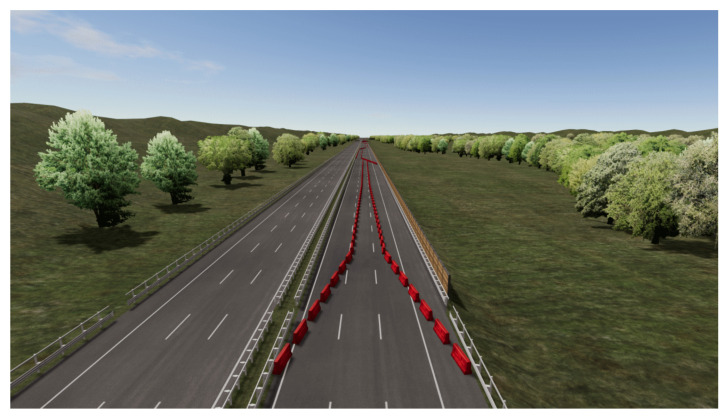
Example of Jersey barriers’ placement to mimic road works; in this figure, a narrow lane followed by a multiple lane change is depicted.

We prepared in advance the simulation of a 20 km-long free highway path in which we placed, every 2 km, a total number of 10 “challenges”. In each challenge, we placed a different sequence of Jersey barriers on the roadway, in order to simulate road works: the resulting challenges were different combinations of multiple lane changes, lane narrowing, multiple lane changes with narrow lanes, and so on; one example of the challenge is shown in Figure 6.

The experiment consisted of driving in three scenes, each with a different sequence of challenges, in order to mimic three different vehicle setups: manual driving (“Manual”), an autonomous vehicle with a “gentle” driving algorithm (“ADAS1”), and an autonomous vehicle with an “aggressive” driving algorithm (“ADAS2”). In the Manual session, the participants had to drive along the roadway respecting speed limits and keeping safely away from the Jersey barriers. In the ADAS1 session, the participants sat on the simulator while the autonomous vehicle performed the maneuvers with a “gentle” approach; in this condition, we limited the vehicle accelerations (longitudinal and lateral) to 8 m/s${}^{2}$ (longitudinal) and 3 m/s${}^{2}$ (lateral): in this way, the autonomous vehicle faces challenges with a prudent driving style. In the ADAS2 session, the participants coped with an autonomous vehicle that faced challenges with an “aggressive” approach; in this condition, the accelerations were limited by the vehicle dynamics. Moreover, the ADAS2 setup was programmed to respect a very narrow distance between the vehicle and the Jersey barriers, with the aim of providing less confidence to the subjects during the test. The sequence of challenges were different in the Manual, ADAS1, and ADAS2 sessions because, otherwise, the participants would have been able to predict the road ahead. Each participant encountered the Manual, ADAS1, and ADAS2 sessions in a randomized sequence. Since the path of each test was 20 km long and the average speed was 120 km/h approximately, the duration of each simulation was roughly 7–10 min.

The model of the vehicle simulated during the experiments was an off-road car, in order to provide to the subjects a vehicle with a low-power-to-weight ratio; in this way, we inhibited, in manual driving, users trying to drive as in a race instead of normal highway driving.

During each session, the data were acquired by the EEG headband from each participant and transmitted to the GUI, on which the data were monitored in real time on the front panel, as shown in Figure 4, and stored in a text file with a 200 Sa/s sampling rate, as described in Section 2.1.3.

#### 2.2.2. Data Pre-Processing

The acquired EEG data are contaminated with different types of artifacts during the experiment; there were artifacts due to the simulator platform movement, which may lead to cable sway, and there were artifacts from subject movements derived from electrical activity due to facial and neck muscles or eye movements, which are common during physical activities [53]. Other physiological artifacts such as eye blink and ECG can also contribute to the brain signal contamination during the experiment. All the acquired data were pre-processed with the EEGLAB toolbox for MATLAB [54] in order to detect and remove artifacts and reconstruct the original signal.

**Figure 7:**
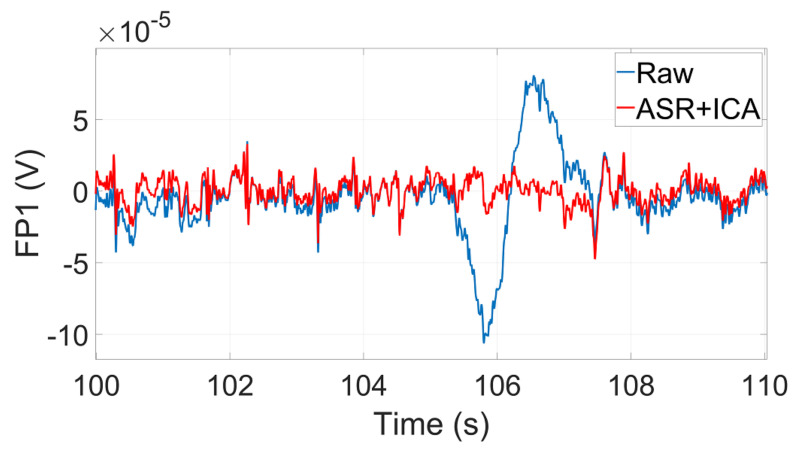
Example of the raw and pre-processed signals.

The pre-processing pipeline consisted of three steps: (1) filtering the data with a basic Finite Impulse Response (FIR) filter with passband edges of [1,45]Hz; (2) removing artifacts with the Artifact Subspace Reconstruction (ASR) algorithm [55]; in this step, we reconstructed the portions of the signals affected by motion artifacts; (3) applying Independent Component Analysis (ICA) [56] method using the RUNICA algorithm to decompose the data into independent components and consequently removing the non-brain components (such as eye blink) from the data. Figure 7 shows an example of the raw (blue) signal and pre-processed (red) signal after the mentioned steps on the FP1 channel of a participant during the ADAS1 session of the experiment; in particular, in Figure 7, the blue line shows an artifact due to the motion of the simulator platform during a lane change.

After artifact removal, we analyzed the signals, evaluating the spectral powers in the frequency bands delta, theta, alpha, beta, and gamma. The results are presented in Section 3.2.

## 3. Experimental Results

In this section, we present the experimental results obtained from two different experiments. The first one is the metrological characterization of the performances of the sensor in terms of gain accuracy, linearity, and bandwidth use and as the reference input, sinusoidal signals and measuring the sensor response at the output. The second experiment showed the results obtained on real EEG traces acquired on the ten volunteers during driving on a simulator.

### 3.1. Metrological Characterization

In this subsection, we characterize the bandwidth and the linearity of the EEG headband experimentally. We connected a waveform generator (Agilent 33220A) to a characterized attenuator, and we acquired the quantity $V_{AD}$ and the output of the generator with an oscilloscope (Keysight DSOX2022A).

Varying the amplitude of the signal, we characterized the gain and linearity (Section 3.1.1); varying the frequency, we characterized the analog front-end bandwidth (Section 3.1.2) and the overall system bandwidth (Section 3.1.3). In particular, the developed GUI allows the insertion of a second-order IIR digital notch filter centered on the power line frequency (50 Hz), so in Section 3.1.3, we quantify the power line rejection and show the overall bandwidth of the system composed of hardware and software.

After acquisition, the data were post-processed according to the “Guide to the expression of Uncertainty in Measurements” (GUM) [45] in order to evaluate the accuracy of the EEG headband.

#### 3.1.1. Linearity Characterization and Resolution

For the linearity and the gain accuracy characterization, the output of a waveform generator provided a sinusoidal voltage $V_{G}$ with 47 linearly spaced amplitudes from 0.1–2.4 $V_{PP}$ with a step size of 50 mV and with a frequency around the center of the bandwidth of the EEG sensor, i.e., at 5 Hz.

We connected a resistive attenuator α (0.1% tolerance of the resistors) to the generator output; the attenuator output impedance was set to 1 MΩ to simulate skin behavior, $\alpha =0.303\times 10^{-3}\left(3\times 10^{-6}\right)$. The output of the attenuator provides the input voltage (referring to Figure 2) $V_{IN}=\alpha V_{G}$ variable from 30 $\mu V_{PP}$ to 730 $\mu V_{PP}$ with a 15 $\mu V_{PP}$ step size; the input of each channel of the EEG sensor was connected to the attenuator. The uncertainty on the input voltage was then calculated according to the GUM:

(3) $$u\left(V_{IN}\right)=\sqrt{{\left[\alpha \cdot u\left(V_{G}\right)\right]}^{2}+{\left[V_{G}\cdot u\left(\alpha \right)\right]}^{2}}$$

In order to estimate the headband accuracy, we evaluated the RMS values of the traces acquired by the oscilloscope on a variable time interval corresponding to 25 periods of the input signal $V_{G}$; for each input amplitude, we acquired 20 RMS values of $V_{G}$ and $V_{AD}$ to perform the type A estimation of uncertainty. Hence, with this setup of acquisitions, we processed a 47 × 20 matrix for each amplified EEG signal $V_{AD}$.

Then, using the least-squares method, we characterized the overall gain $G=G_{1}\cdot G_{2}$ using as the input the voltage vector ($V_{IN}$) and as the output the mean of the 20 RMS readings ($\bar{V_{AD}}$) calculated for each amplitude of the input signal [37,47]:

(4) $$G=\frac{\langle V_{IN}\bar{V_{AD}}\rangle -\langle V_{IN}\rangle \langle \bar{V_{AD}}\rangle }{\langle V_{IN}^{2}\rangle -{\langle V_{IN}\rangle }^{2}}$$

We evaluated the combined uncertainty of the output voltage $u(\bar{V_{AD}})$ for each channel taking into account the type A estimation $u_{A}$ (as the standard deviation of the sample means over the 20 readings of the RMS) and the type B estimation $u_{B}$ extracted from the manual of the oscilloscope:

(5) $$u\left(\bar{V_{AD}}\right)=\sqrt{{\left[u_{A}\left(\bar{V_{AD}}\right)\right]}^{2}+{\left[u_{B}\left(\bar{V_{AD}}\right)\right]}^{2}}$$

We recall that the vector $V_{IN}$ was composed of 47 test amplitudes and that the vector $\bar{V_{AD}}$ was obtained by calculating the mean of 20 readings for each output amplitude. Naming with subscript *i* the input and output voltages at the $i_{th}$ amplitude and using (3), (4), and (5), we estimated the gain uncertainty as:

(6) $$u\left(G\right)=\sqrt{\sum_{i=1}^{47}{\left(\frac{\partial G}{\partial V_{IN,i}}u\left(V_{IN,i}\right)\right)}^{2}+\sum_{i=1}^{47}{\left(\frac{\partial G}{\partial \bar{V_{AD,i}}}u\left(\bar{V_{AD,i}}\right)\right)}^{2}}$$

**Figure 8:**
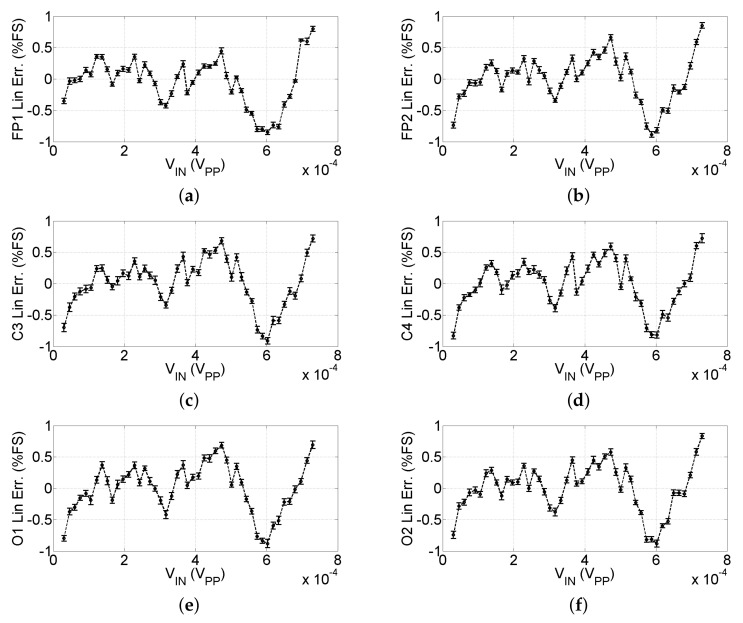
Linearity of the headband channels; error bars represent the uncertainty on the linearity. (**a**–**f**) linearity of the channels Fp1, Fp2, C3, C4, O1, and O2, respectively.

After characterization, the gains of the six channels resulted in G=4210±35. Using the gain calculated in (4), we evaluated the deviation from the linear regression. In Figure 8, we show the linearity error normalized to the Full Scale (FS) of each input.

Referring to Figure 8, the non-linearity for the six channels resulted in roughly 0.8% FS, corresponding to 6μV; error bars represent the uncertainty estimated in (3) and (5).

As reported in Section 2, the A/D resolution was increased to 14 bit using the oversampling technique; since the full scale of the sensor was ±350μV, this corresponds to 43nV.

The noise voltage density of the chosen instrumentation amplifier was, by the datasheet, $35\;\;nV/\sqrt{Hz}$, so integrating over the sensor bandwidth, the analog circuitry introduces the noise $\sigma_{A}=230\;\;nV$ (comparable to the resolution without oversampling); combining this quantity with the quantization noise (i.e., $\sigma_{Q}=3.3/\left(G\cdot 2^{12}\cdot \sqrt{12}\right)\approx 60\;\;nV$), we obtained the overall noise introduced by the sensor as $\sqrt{\sigma_{A}^{2}+\sigma_{Q}^{2}}=240\;\;nV$ without oversampling. Taking into account the improvement shown in (2), we can quantify the noise after oversampling in 60nV, and the Effective Number of Bits (ENOB) of the sensor would result in ENOB=13.51 bits.

#### 3.1.2. Analog Bandwidth Characterization

For the characterization of the analog front-end bandwidth, the waveform generator provided a sinusoidal voltage with amplitude 2.4 $V_{PP}$ (corresponding to $V_{IN}=730\;\;\mu V_{PP}$) and frequencies spanning in the range [0.1,1000] Hz with 81 logarithmically spaced steps; the output of the generator was connected to the same attenuator described in the previous subsection.

With the same procedure shown in Section 3.1.1, we acquired the outputs for each frequency step in a time window corresponding to 10 periods of the input signal; in this way, we computed the gain G(jω) on an integer number of periods (coherent sampling).

**Figure 9:**
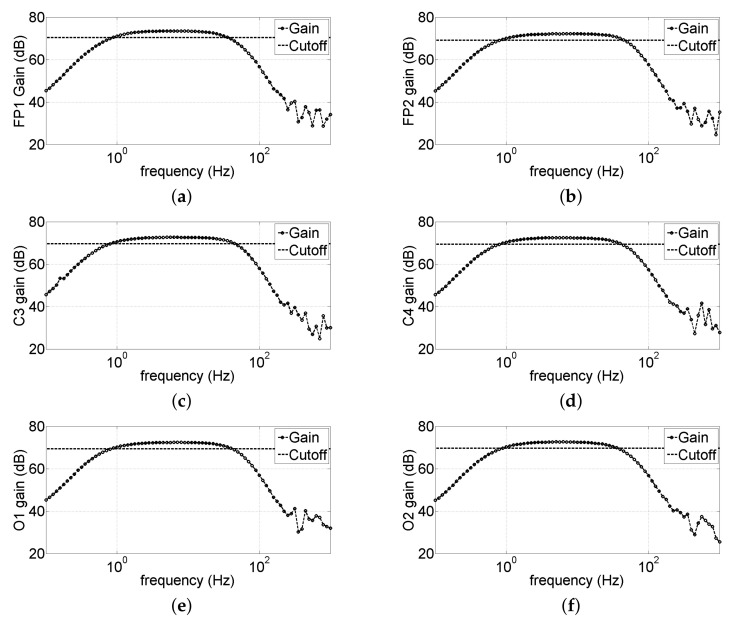
Bandwidth characterization of the analog front-end of the headband; the horizontal line represents the cutoff limit. (**a**–**f**) Bandwidth of the channels Fp1, Fp2, C3, C4, O1, and O2, respectively.

Figure 9 shows the gain modulus of all six channels; the upper cutoff frequency resulted in being 44 Hz, while the lower cutoff was 0.8 Hz.

#### 3.1.3. Overall Bandwidth Characterization

**Figure 10:**
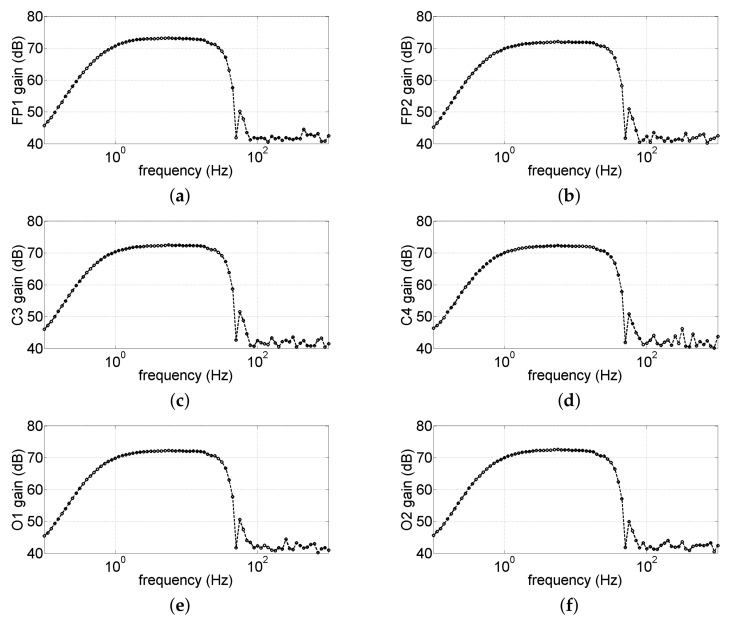
Bandwidth characterization of the entire system with the digital notch IIR filter. (**a**–**f**) Bandwidth of the channels Fp1, Fp2, C3, C4, O1, and O2, respectively.

As described in the previous section, the GUI allows the optional insertion of a second-order IIR notch filter to remove power line disturbance. In order to characterize the effects of the notch filter, with the GUI, we logged the data obtained during the characterization described in Section 3.1.2 and estimated the bandwidth of the data acquired by the GUI when we enabled the notch filter. Figure 10 shows the frequency response of the data plotted on the GUI; from Figure, it is evident that the power line (corresponding to 50 Hz), thanks to the digital notch filter insertion, was attenuated by 30 dB.

### 3.2. Results of EEG Signals on a Driving Simulator

**Figure 11:**
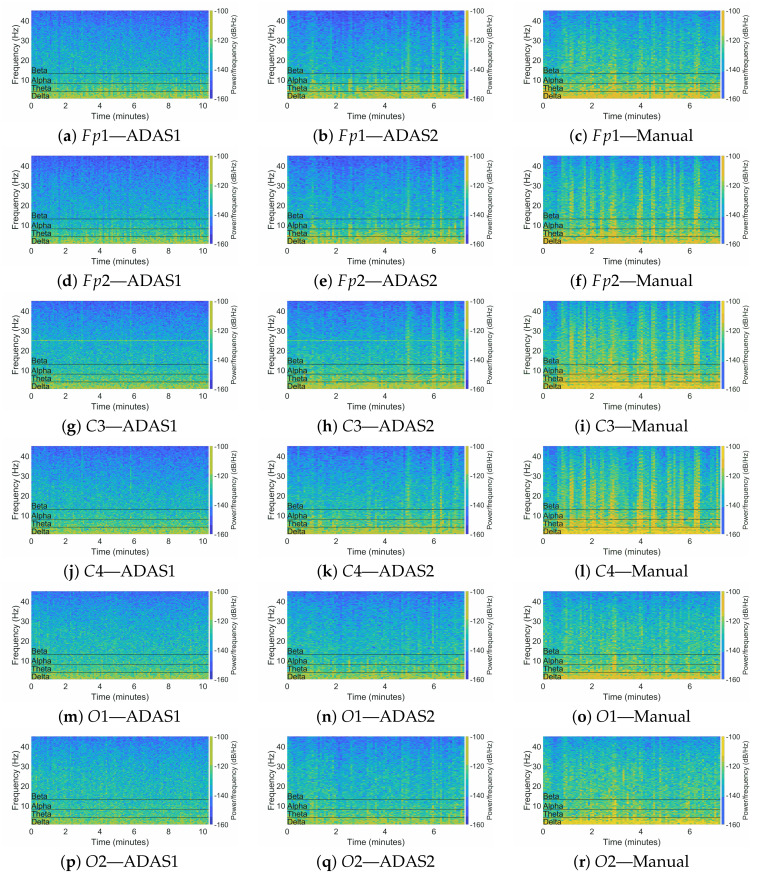
Spectrograms of the six channels extracted after the experiment sessions. (**a**–**c**) Fp1 channel; (**d**–**f**) Fp2 channel; (**g**–**i**) C3 channel; (**j**–**l**) C4 channel; (**m**–**o**) O1 channel; (**p**–**r**) O2 channel. Pictures on the left column refer to the ADAS1 session, in the middle column refer to ADAS2 session, and in the right column refer to the Manual session.

The distance covered in all the experiment sessions was 20 km; this length was designed in order to obtain a rough duration of 10 min per session, considering an average speed of 120 km/h. Depending on the speed of each driver, the duration of the acquired data was in the range of 7–10 min. Figure 11 shows the time–frequency analysis on the six channels during the three sessions relative to one of the drivers.

From a qualitative point of view, the ADAS1 session was characterized by low spectral components in any frequency band and for the entire duration of the session; this may represent low stress during autonomous smooth driving. In the ADAS2 session, instead, we saw that there were some higher components in theta and alpha bands, and there were also some significant components in the beta band; this may be indicative of a less-relaxing state of the driver during ADAS2. As a preliminary analysis, we observed that the ADAS2 session manifested slightly higher EEG activity with respect to ADAS1. During the Manual session, instead, it was possible to see strong components in the beta band, which was almost absent in ADAS1 and was lower in ADAS2.

The EEG beta power is known to be highly correlated with the cortisol hormone level in the body, which increases in response to stress [57]. Moreover, beta power is known to be an indicator of attention, concentration, and anxiety [42,58]. Numerous studies selected the power increase of the beta waves to identify stress [59,60,61,62] and the power decrease of the beta waves to identify mental fatigue and drowsiness [63,64,65]. For these reasons, in the present paper, we focused our attention on the power of beta waves to identify stress. To quantify the beta power, we evaluated the Power Spectral Density (PSD) of the signals over a time window of duration 4 s, using as a window function the von Hann window with 50% overlap between windows.

**Figure 12:**
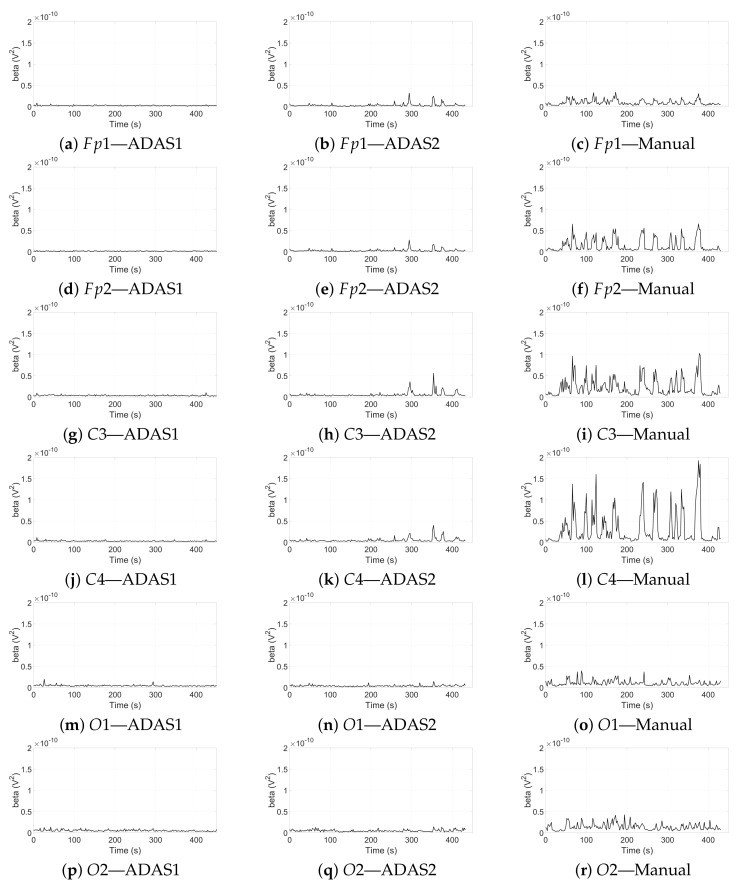
Beta wave powers relative to the six channels calculated after processing. (**a**–**c**) Fp1 channel; (**d**–**f**) Fp2 channel; (**g**–**i**) C3 channel; (**j**–**l**) C4 channel; (**m**–**o**) O1 channel; (**p**–**r**) O2 channel. Pictures on the left column refer to the ADAS1 session, in the middle column refer to the ADAS2 session, and on the right column refer to the Manual session.

Figure 12 shows the powers in the beta band during the three sessions relative to the spectrograms in Figure 11; as can be seen, the beta power resulted in being low during ADAS1, slightly higher during ADAS2, and significantly higher during Manual. This suggests that the stress on the driver was by far higher in the Manual session. This result is consistent with our previous findings [9], where the EDA activities and mean heart rate (stress indicators) were shown to be higher in manual driving with respect to autonomous driving. In Table 3, we report the mean values of the beta power on the six channels for all the drivers, during the three sessions of the experiment.

**Figure 13:**
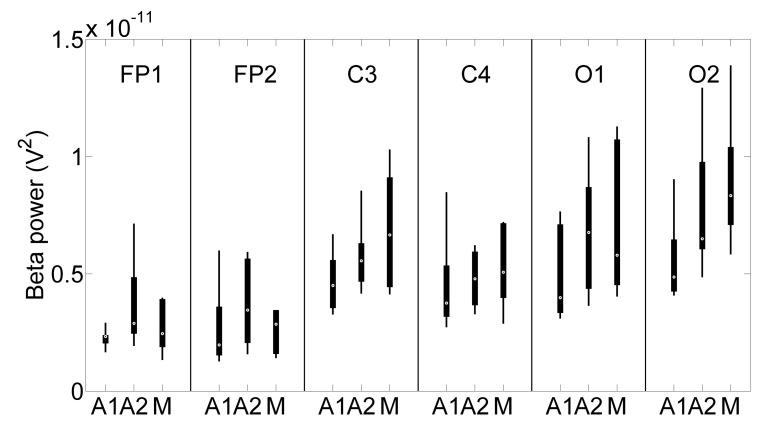
Box plot of the beta powers relative to the ten volunteers. Markers represent the medians, boxes 25–75 percentiles, and whiskers 5–95 percentiles. The tendency of increasing beta power from ADAS1 to ADAS2 and from ADAS2 to Manual is noticeable.

Figure 13 shows the box plot of the data reported in Table 3. Markers represent the medians, boxes the 25 to 75 percentiles, and whiskers the percentiles from 5 to 95. In the figure, it is possible to see a quite evident tendency in almost all the channels of low beta power during ADAS1, medium power in ADAS2, and high power in Manual. The tendency of increasing beta powers from ADAS1 to ADAS2 or from ADAS1 to Manual was expected by design, since ADAS1 was configured to provide confidence to the users, putting them in a relaxed state due to a prudent autonomous vehicle; on the other hand, we expected that ADAS2 would have been the most stressful session for the users, since we configured ADAS2 to perform sudden maneuvers with high lateral acceleration and with closer distances from the Jersey barriers. However, in Figure 13, we observe a slight trend of increasing beta powers from ADAS2 to Manual; this would suggest that the users during Manual driving had to bear higher mental load with respect to ADAS2 because they had to be attentive to the barriers and follow the road cautiously to avoid accidents. This result is in accordance with other studies, where higher mental load provoked by harder driving tasks was observed by means of EEG activity [66]. Since several box plots in Figure 13 are overlapping, we must consider if these results assume statistical significance.

In order to quantify the statistical significance of the differences between ADAS1, ADAS2, and Manual, we performed the non-parametric statistical Wilcoxon signed rank test; we report the probability of the null hypothesis in Table 4. If the probability is lower than 0.05, then there is a significant statistical difference between two experimental setups.

From Table 4, it emerges that beta power in ADAS1 resulted in being significantly lower than the ADAS2 one in the channels Fp1 and O2; we noticed that in the channel O1, p=0.06, very close to statistical significance; the beta power of ADAS1 resulted in being significantly lower than the Manual one in the channels C3, O1, and O2; the ADAS2 beta power instead resulted in being lower than the Manual one, but with poor statistical significance. A qualitative insight of this can also be found in Figure 13; qualitatively comparing the boxes relative to ADAS2 and Manual, it is possible to observe that in all channels, ADAS2 and Manual manifest strongly overlapped boxes, i.e., the statistical data among the users belong to similar distributions.

## 4. Discussion

Regarding the experimental characterization of the sensor, we can assert that the performances of the developed headband respected the expectations in terms of the accuracy and bandwidth; in particular, we recall that the non-linearity of all channels was 0.8%FS, corresponding to 6 μV, and the bandwidth was in the range [0.8, 44] Hz with a power line rejection of 30 dB thanks to the IIR digital filter implemented on the developed GUI.

Regarding the test drive on the simulator performed by the ten volunteers, the aim of the experiment was to demonstrate that the EEG headband was able to discriminate the sensation of confidence perceived by the drivers during manual driving with respect to autonomous vehicles with gentle or aggressive algorithms. To do this, we focused our study on the power of the beta waves because in the scientific literature, it is evaluated as an indicator of discomfort. Actually, in the first phase of designing the experiment, we would have expected that ADAS2 would have been perceived as the most stressful experiment, while ADAS1 was designed to be the least stressful; however, as shown in Figure 13, the evidence showed that ADAS1 manifested the lowest beta wave power, as expected, but the Manual experiment was perceived, on average, as the most expensive in terms of mental load.

As reported in Table 4, we see that the O1 and O2 channels provided a statistically significant difference between ADAS1 vs. ADAS2 and ADAS1 vs. Manual; even if ADAS2’s beta power was lower than the Manual beta power, the difference turned out to be not statistically significant, since the *p*-values in the last column of Table 4 are higher than 0.05. For this reason, we can just assert that Manual was slightly more stressful than ADAS2, but with quite low statistical significance.

## 5. Conclusions

We presented the design of a wearable EEG headband for the measurement of stress-related brain activity during driving. The EEG data are sent via WiFi to a laptop and the sensor is battery operated, so the headband is completely wireless in order to have high wearability. The characterization showed high-level performances in terms of linearity, resolution, and battery life. The metrological characterization of the sensor is of paramount importance in order to understand the accuracy of the used sensor, especially if the data are then processed through ML algorithms. Most of the literature works in fact made use of commercial devices where the measurement error is not provided in the sensors’ manuals.

We tested the proposed sensor, setting up an experiment that involved ten volunteers; the aim was comparing three different scenarios: manual driving, autonomous vehicle with “gentle” algorithm (ADAS1), and autonomous vehicle with “aggressive” algorithm (ADAS2). Processing the EEG data and evaluating the beta wave power, we observed that Manual driving revealed the highest stress on drivers; ADAS2 manifested higher stress than ADAS1 and slightly lower than Manual; ADAS1 instead presented the lowest stress on drivers with good statistical significance, especially on channels O1 and O2.

As future work, we will integrate the EEG data with the data coming from the ECG and EDA sensors that we developed in previous works. Thanks to the total control of the transmission protocol, in fact, the sensor allows the simultaneous acquisition of different bio-signals using a common timebase, having a high-accuracy time alignment.

## Figures and Tables

**Table 1:** Brain waves and their description.

Wave Type	Frequency Range (Hz)	Description
Delta	0.5–4	Deep sleep
Theta	4–8	Sleep, meditation, concentration
Alpha	8–12	Relax, reflection
		Decreasing amplitude with anxiety
Beta	12–30	Alert, focused
		Increasing amplitude with
		stress, excitement, high mental activity
Gamma	>30	Focus, sensory processing
		Increasing amplitude with anxiety

**Table 2:** Commercial EEG headbands’ specifications and comparison with the present paper.

Model	Channel #	Sample Rate (Hz)	Resolution (nV)	Linearity	Bit #	Battery (h)
Emotiv Epoc	14	128	510	N/A	14	9
Neurosky Mindwave	1	512	N/A	N/A	12	8
Interaxon Muse 2	4	220–500	488	N/A	12	5
Present paper	6	200	43	0.8%	14	10

**Table 3:** Mean beta powers for every channel and scenario relative to each volunteer.

**Mean beta power for ADAS1 session (** $\mu V^{2}$ **)**						
**Subject #**	Fp1	Fp2	C3	C4	O1	O2
1	2.33	1.99	3.27	3.97	3.34	4.68
2	4.63	5.99	6.69	7.12	7.66	6.90
3	2.39	1.68	3.54	2.90	4.37	5.05
4	2.91	3.60	4.83	3.58	3.53	4.25
5	2.39	1.95	5.34	5.35	7.40	6.46
6	1.66	1.51	4.18	2.72	3.10	4.63
7	2.12	1.53	6.42	8.48	3.98	5.24
8	2.33	2.08	3.92	3.26	3.14	4.10
9	2.04	1.27	3.39	3.17	7.11	9.03
10	1.20	4.37	5.59	3.92	3.99	4.08
**Mean beta power for ADAS2 session (** $\mu V^{2}$ **)**						
**Subject #**	Fp1	Fp2	C3	C4	O1	O2
1	2.56	2.73	4.67	3.91	9.06	6.63
2	4.86	5.81	5.40	6.22	7.67	9.77
3	3.21	3.05	5.71	4.85	3.91	4.86
4	4.90	4.24	8.55	4.73	5.19	5.55
5	2.45	2.05	6.30	5.42	8.70	6.31
6	1.93	1.61	4.17	3.67	4.37	7.10
7	7.14	5.65	10.68	15.81	6.45	6.05
8	3.75	3.86	6.24	3.62	3.64	17.19
9	2.54	1.58	4.17	3.28	10.83	12.94
10	1.93	5.94	5.34	5.95	7.07	6.37
**Mean beta power for Manual session (** $\mu V^{2}$ **)**						
**Subject #**	Fp1	Fp2	C3	C4	O1	O2
1	2.26	3.13	10.30	4.42	7.41	8.29
2	2.53	2.58	6.80	5.72	5.14	8.71
3	9.64	14.93	22.75	31.55	10.73	13.89
4	2.38	1.41	9.11	7.21	6.45	6.07
5	3.93	3.27	8.55	7.16	11.28	10.41
6	1.33	1.41	4.25	2.98	4.04	7.09
7	2.84	2.38	5.99	6.92	4.61	8.38
8	3.98	3.46	4.44	4.34	4.45	7.10
9	1.81	1.59	4.12	2.88	11.24	11.07
10	1.88	7.55	6.51	3.98	4.52	5.83

**Table 4:** Wilcoxon test probabilities. The values with p≤0.05 are in bold.

Channel	Wilcoxon Test Probability		
	**ADAS1 vs. ADAS2**	**ADAS1 vs. Manual**	**ADAS2 vs. Manual**
Fp1	**0.05 **	0.52	0.31
Fp2	0.16	0.47	0.57
C3	0.12	**0.02**	0.38
C4	0.21	0.18	0.68
O1	0.06	**0.02**	0.79
O2	**0.02**	**0.003**	0.34
